# Hmga2 translocation induced in skin tumorigenesis

**DOI:** 10.18632/oncotarget.16272

**Published:** 2017-03-16

**Authors:** Yong Li, Xiang-ying Pi, Kelsey Boland, Sonali Lad, Kelly Johnson, Catherine Verfaillie, Rebecca J. Morris

**Affiliations:** ^1^ The Hormel Institute University of Minnesota, Austin, MN 55912, United States; ^2^ Department Development and Regeneration, Stem Cell Institute, KU Leuven, Leuven 3000, Belgium

**Keywords:** Hmga2, keratinocytes, panobinostat, ROCK, skin tumorigenesis

## Abstract

Hmga2 protein, a transcription factor involved in chromatin architecture, is expressed chiefly during development, where it has many key biological functions. When expressed in adult tissues from in various organs, Hmga2 is always related to cancer development. The role of Hmga2 in skin tumorigenesis is, however, not yet understood. We demonstrated that Hmga2 can be found in non-transformed epidermis, specifically located to the membrane of keratinocytes (KCs) in epidermis. *Ex vivo* culture of KCs and development of skin carcinomas in DMBA and TPA mouse models was associated with translocation of the Hmga2 protein from the membrane into the nucleus, where Hmga2 induced its own expression by binding to the Hmga2 promoter. Panobinostat, an HDAC inhibitor, downregulated Hmga2 expression by preventing Hmga2 to bind its own promoter, and thus inhibiting Hmga2 promoter activity. Hmga2 translocation to the nucleus could in part be prevented by an inhibitor for ROCK1. Our findings demonstrate that upon program of benign papilloma to malignant cSCC of skin tumorigenesis, Hmga2 translocates in a ROCK-dependent manner from the membrane to the nucleus, where it serves as an autoregulatory transcription factor, causing cell transformation.

## INTRODUCTION

Cutaneous squamous cell carcinoma (cSCC), the second most frequent skin cancer, often arises from the progression of benign lesions [1]. This progression is believed to be due to sequential DNA mutations in oncogenes and tumor suppressor genes including TP53, NOTCH1 and CDKN2A [2]. Protein mislocalization also plays an important role in cancer initiation and progression. For instance, translocation of NF-kB and beta-catenin from the cytoplasm to nucleus is frequently seen in cancer cells [3]. However, the molecular underpinnings that drive cSCC initiation, progression, and metastasis are still not fully understood, a prerequisite for the development of better therapeutic options [4].

High mobility group AT-hook 2 (Hmga2) is a member of the HMG family of proteins. Hmga2 was first described in 1991 [5] as a nuclear architectural protein that interacts with the minor groove of many AT-rich promoters and enhancers through AT-hooks [6]. The molecule is widely expressed in undifferentiated cells during embryogenesis, becomes more restricted as fetal development progresses [7, 8] and is limited to the mesenchyme in adults [9]. The promoter of Hmga2 contains Sp1, Sp3 and RUNX1 binding sites [10, 11], and the 3′ untranslated region (UTR) contains bindings sites from multiple miRNAs, including miRlet-7, miR10A and miR21 [12, 13]. Hmga2 levels were shown to be regulated by ROCK inhibitors, causing shortening of the poly (A) tail via let-7 [14]. In addition, histone deacetylase inhibitors (HDACI) such as panobinostat, Sirtuin 6 and trichostatin A significantly reduced the steady-state level of Hmga2 through let-7, Sp1 and Sp3 [4, 10, 15].

Hmga2 also plays crucial roles in neural stem cell [16] and hair follicle stem cell [17] selfrenewal, as well as in adult somatic reprogramming [18]. In addition, Hmga2 plays a role in a variety of malignant tumors, including colorectal, breast, lung, prostate and bladder cancer as well as melanoma [21–26]. However it is not known if the role of Hmga2 during cSCC initiation and promotion.

Given its critical roles in several other developmental systems including malignancies we therefore investigate Hmga2 function in the context of the well-defined multistage model of cutaneous carcinogenesis in mice. We made the surprising discovery that Hmg2 was expressed in the cell membranes in normal epidermis and freshly isolated epidermal keratinocytes. Hmga2 rapidly translocated to the nucleus upon cell culture and during development of cSCCs. We examined the molecular mechanism regulating this translocation *in vitro*, *ex vivo*, and *in vivo*. Together, these studies demonstrated that Hmga2 translocation functions in an auto-regulatory loop associated with the induction of cSCCs.

## RESULTS

### Hmga2 expression and cellular localization in keratinocytes and skin tumor development

**Figure 1:**
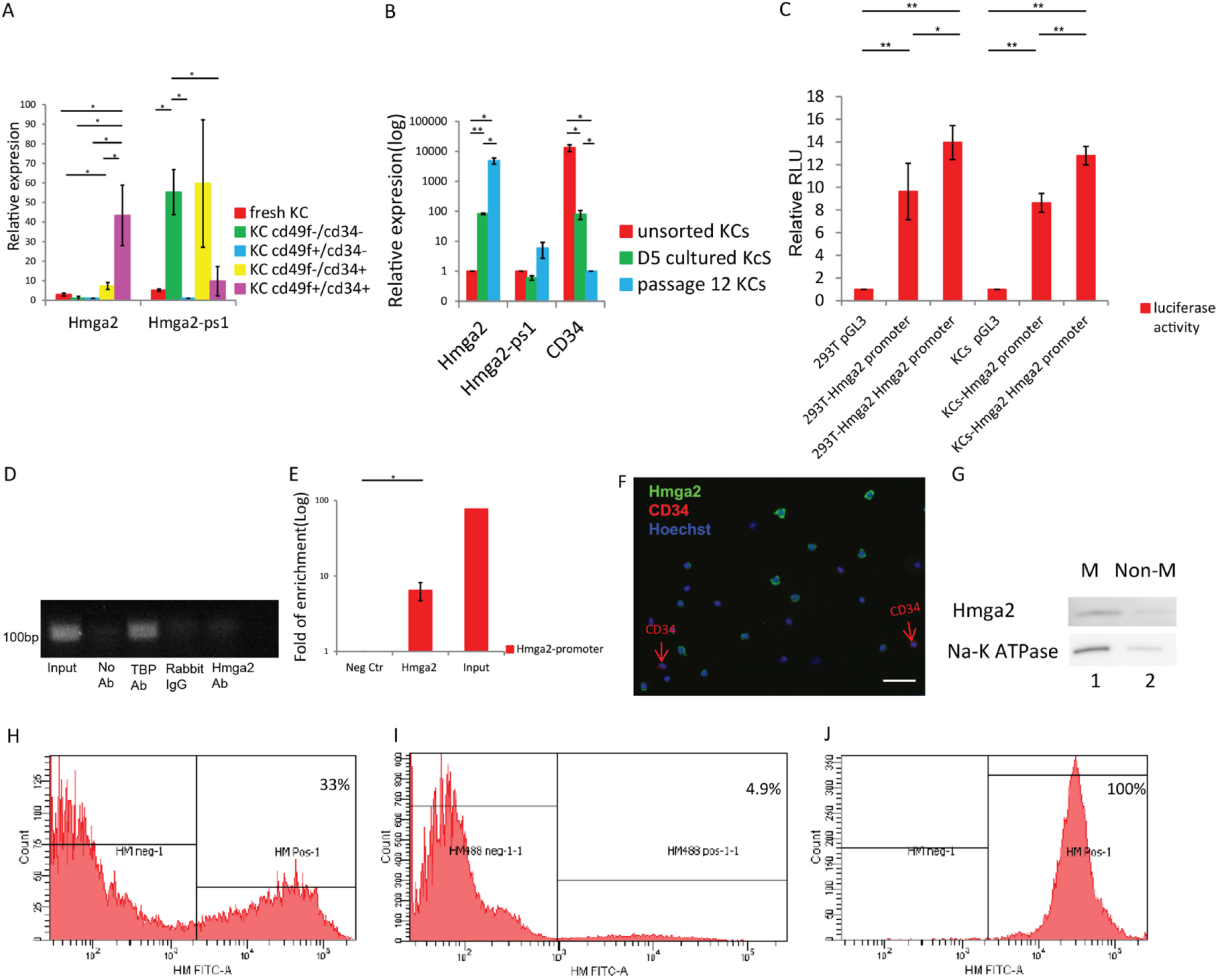

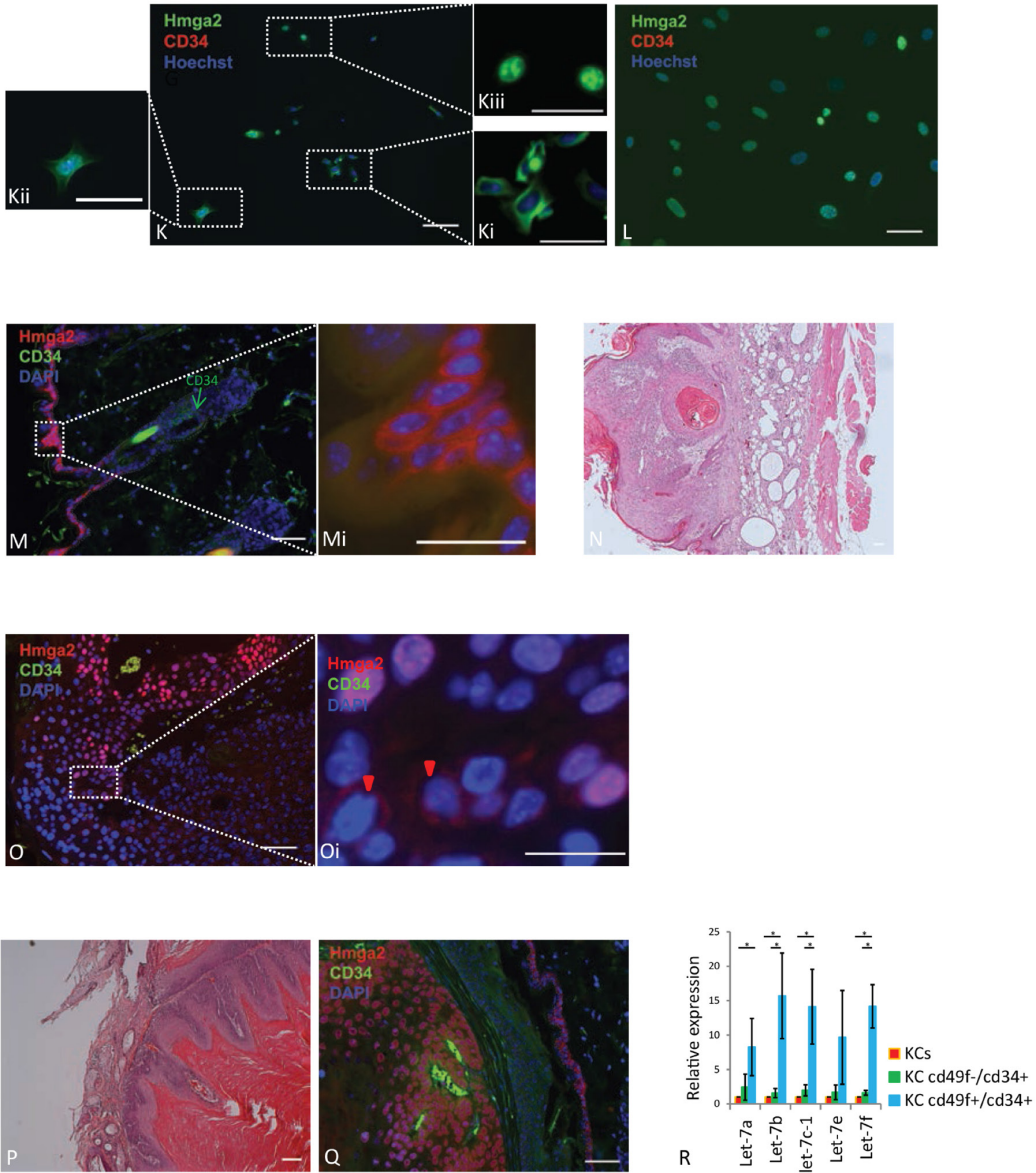
Hmga2 can be expressed as a non-nuclear protein in the adult organs (**A**) Hmga2 and Hmga2-ps1 mRNA levels were assessed by qRT-PCR in unsorted and sorted KCs by CD34 and CD49f antibodies (*n* = 3). (**B**) Hmga2, Hmga2-ps1 and CD34 mRNA levels were assessed by qRT-PCR in unsorted KCs, day5 cultured KCs and long-term cultured KCs (passage 12). (**C**) 293T and KCs cell lines were transfected with pGL3, full-length Hmga2 promoter-luciferase construct (pGL3-Hmga2 promoter), and luciferase assays were performed 48 hours after transfection. Co-expression was conducted with Hmga2 expression vectors. Error bars indicate standard deviation (*n* = 3). (**D**) Agarose gel electrophoresis for enriched GAPDH promoter at 30 cycles. (**E**) ChIP assay for Hmga2 binding to the Hmga2 promoter. (**F**, **K**, **L**) unsorted (**F**), short-term cultured (day 5) (**K**) and long-term cultured KCs (passage 12) (**L**) were fixed, and subjected to immunofluorescence using Hmga2 and CD34 antibodies. Nuclei were stained using Hoechst33342, Hmga2 green, CD34 red (*n* = 3). Ki-Kiii. higher magnification showing the expression of Hmga2 at membrane, entire cells and nucleus. (**G**) Hmga2 protein expression in KCs from membrane and non-membrane. Membrane (M) and non membrane (non-M) fractions containing equal amounts of protein. (**H**–**J**) Hmga2 protein expression determined by flow cytometry. (**H**), unsorted KCs; (**I**), passage 12 KCs; (**J**) permeabilized passage 12 KCs. (**M**, **O**, **Q**) Normal skin tissue (**M**), papilloma (**O**) and carcinoma tissue (**Q**) were fixed, and subjected to immunofluorescence using Hmga2 and CD34 antibodies. Nuclei were stained using DAPI, Hmga2 red, CD34 green (*n* = 3). Mi. Higher magnification showing the expression of Hmga2 in the membrane in normal skin tissue. Oi. Higher magnification showing the expression of Hmga2 in membrane (red arrowhead) and nucleus in papilloma. (**N** and **P**) Hematoxylin and Eosin (H&E) staining for skin tissue from papilloma and carcinoma. R. let-7 family RNA were assessed by qRT-PCR in unsorted and sorted KCs by CD34 and CD49f antibodies (*n* = 3). The scale bar size 50 mm. *P < 0.05. **P < 0.01.

We isolated keratinocytes (KCs) from mouse skin and isolated KC stem cells based on CD49f and CD34 expression. RT-qPCR analysis demonstrated that Hmga2 transcript levels were significantly higher in CD49f+/CD34+ compared with CD49f+/CD34− cells; while transcript levels for the Hmga2-pseudogene (Hmga2-ps1) were not enriched in the CD49f+/CD34+ compared with the CD49f+/CD34− fraction (Figure 1A). We then cultured KCs for 5 days and up to 12 passages. Hmga2 transcripts significantly increased in KCs upon culture (Figure 1B, Supplementary Figure 1A), while CD34 expression decreased, and expression of Hmga2-ps1 and Hmga1 was unchanged (Figure 1B, Supplementary Figure 1B and 1C).

Because Hmga2 transcript levels increased significantly during KC culture, we determined the Hmga2 promoter activity and whether Hmga2 might regulate Hmga2 promoter activity. For this we co-transfected the full-length promoter of Hmga2 upstream of a luciferase cassette together with Hmga2 cDNA in 293T and KCs. Luciferase activity increased to 44% and 48%, respectively in 293T cells and KCs (Figure 1C). We further examined if Hmga2 directly binds to the Hmga2 promoter (base pairs [− 800 to + 197] relative to the TSS) by ChIP followed by qPCR. These studies demonstrated that compared with rabbit IgG, anti-Hmga2 ChIP significantly enriched the Hmga2 promoter region in cultured keratinocyte cells (Figure 1D, 1E).

As Hmga2 appeared to play an autoregulatory function following culture of KCs, we next determined the location of Hmga2 in fresh and cultured KCs. Unsorted and cultured KCs were immunostained with Hmga2 and CD34 antibodies. Hmga2 was detected in the cell membrane of 44.2 ± 9.5% % of KCs, which did not co-localize with CD34 protein staining, (Figure 1F). Western blot of membrane and non-membrane fractions further confirmed the membranous vs non-membrane location of Hmga2 protein in freshly isolated KCs (Figure 1G). Flow cytometry showed that more than 30% KCs expressed membrane-specific Hmga2 (Figure 1H), Hmga2 expressed in all permeabilized passage 12 KCs while only less 5% passage 12 KCs expressed Hmga2 without permeabilized (Figure 1I, 1J). After KCs were cultured for 5 days, Hmga2 protein could be detected both in the cell membrane (Figure 1Ki), throughout the entire cell (Figure 1Kii) and in the nucleus (Figure 1Kiii), while Hmga2 protein could only be detected in the cell nucleus in KCs (that stained positive for keratin 14, Supplementary Figure 2A) at 12 passage (Figure 1L). Thus, upon culture of KCs, Hmga2 translocated from the membrane to the nucleus, where it functions in an autoregulatory loop. We also assessed the presence of Hmga2 and CD34 in normal mouse skin. CD34, but not Hmga2 staining was seen in the hair follicle bulge, while Hmga2 staining was found in the epidermis, where it was located in the membrane of epidermal epithelial cells (Figure 1M). Hmga2 protein was also detected in the cell membrane of esophageal epithelial cells (Supplementary Figure 2B and 2C).

Interestingly, when Hmga2 staining was performed on mouse skin papilloma tissue (Figure 1N), Hmga2 was also found in the nucleus in most cells (Figure 1O) while Hmga2 was cell membrane associated in a few cells (Figure 1Oi). In carcinomas (Figure 1P), Hmga2 was found only in the nucleus (Figure 1Q) of K14 positive cells (Supplementary Figure 2D and 2E), indicating that Hmga2 also translocated from the membrane to the nucleus during carcinoma development.

To understand the discrepancy between Hmga2 transcript and protein levels in CD34+ KC stem cells, we hypothesized that miR let-7 members, known to bind to the 3′UTR of Hmga2, might inhibit protein expression [12]. Therefore, RT-qPCR was performed to measure levels of let-7 miRNAs. We found that all miRNAs from the let-7 family assessed were significantly more expressed in the CD49f+/CD34+ cell fraction compared with unsorted KCs and the CD49f+/CD34− fraction (Figure 1R), suggesting that Hmga2 protein levels in keratinocyte stem cells selected from KCs by FACS, and located in the hair follicle bulge, might be inhibited by let-7 miRNAs. Thus, Hmga2 protein levels in hair follicle stem cells may be regulated by Let7-miRNAs.

### Panobinostat affects keratinocyte proliferation as well as transcription and cellular localization of Hmga2, during skin tumor development

**Figure 2:**
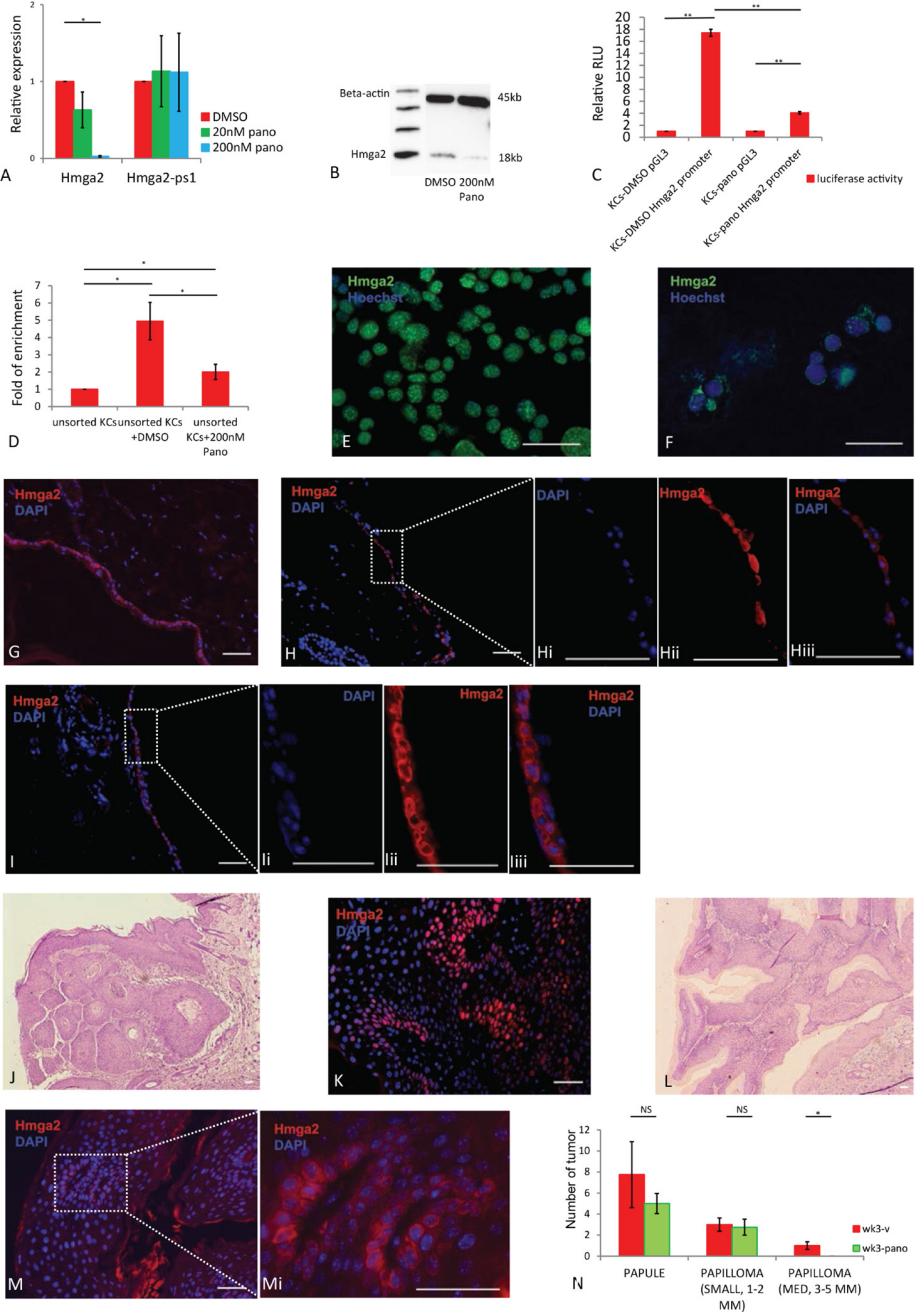
Hmga2 translocation to nucleus can be inhibited by panobinostat (**A**) Hmga2, Hmga2-ps1 mRNA levels were assessed by qRT-PCR in KCs treated with 20 nM, 200 nM panobinostat. (**B**) Analysis of Hmga2 expression by western blot in KCs after treatment with 200 nM panobinostat. (**C**) Hmga2 promoter activity in KCs after treatment with panobinostat. KCs cell lines treated with DMSO or 200 nM panobinostat and were transfected with pGL3, full-length Hmga2 promoter-luciferase construct (pGL3-Hmga2 promoter), and luciferase assays were performed 48 hours after transfection. (**D**) ChIP assay for Hmga2 binding to the Hmga2 promoter. KCs cells were cultured with DMSO or 200 nM panobinostat for two days. Cells were harvested and ChIP was performed with an isogenic or anti-Hmga2 antibodies. Enrichment for the Hmga2 promoter in the precipitated DNA was analyzed by qPCR using primers that flank the distal promoter region. Results from triplicate experiments are shown as fold change of DNA enrichment. (**E** and **F**) KCs treated with DMSO and panobinostat, and subjected to immunofluorescence using Hmga2 antibodies. Nuclei were stained using Hoechst33342, Hmga2 green (*n* = 3). (**G**, **H** and **I**) *Ex vivo* cultured skin tissue treated with DMSO and panobinostat were fixed, and subjected to immunofluorescence using Hmga2 antibodies. Nuclei were stained using DAPI, Hmga2 red. Hi-Hiii. Higher magnification showing the expression of Hmga2 in the nucleus and cytoplasm. Ii-Iiii. Higher magnification showing the expression of Hmga2 in the cytoplasm. (**K** and **M**) Papillomas from mouse treated with vehicle and panobinostat were fixed, and subjected to immunofluorescence using Hmga2 antibodies. Nuclei were stained using DAPI, Hmga2 red. (**J** and **L**) H&E staining for papillomas from mice treated with vehicle and panobinostat. (**N**) Papules and papillomas number from SKH mouse after treated vehicle and panobinostat at 3 weeks. The scale bar size 50 mm. * *P* < 0.05. **P < 0.01.

HDACIs such as panobinostat can inhibit Hmga2 gene expression in NIH3T3, F9, Hela, liver cancer cell lines and human cord blood-derived multipotent stem cells [10, 15, 27]. To determine if panobinostat is capable of regulating endogenous Hmga2 gene expression in the skin, we performed Hmga2 RT-qPCR and protein analysis following treatment of freshly isolated KCs cultured with either DMSO alone or 20 nM and 200 nM panobinostat, dissolved in DMSO. Hmga2 levels were significantly reduced after treatment with 200nM panobinostat for one day compared with DMSO control cultures (Figure 2A, 2B). As expected, panobinostat significantly induced levels of acetyl histone 3 (Supplementary Figure 3A). To further demonstrate that panobinostat regulated Hmga2 gene transcription, KCs were pre-treated with the transcription inhibitor, actinomycin D (Act-D), for 30 min prior to addition of either panobinostat or DMSO [10]. RT-qPCR analysis demonstrated that addition of Act-D to Panobinostat further decreased Hmga2 mRNA levels (Supplementary Figure 4A) suggesting that panobinostat inhibits Hmga2 gene transcription, but not to the same extent as Act-D. We also assessed expression levels of the let-7 miRNA family in response to panobinostat. As expected, no significant changes were found, consistent with the notion that let-7 miRNAs regulate translation and not transcription of Hmga2 (Supplementary Figure 3B). To further substantiate the decreased transcription of Hmga2 in response to panobinostat, we repeated the Hmga2 luciferase promoter studies and Hmga2 ChiP-Seq studies in the presence of panobinostat. We found that the Hmga2 promoter activity was significantly repressed after treatment with panobinostat compared with DMSO treatment (Figure 2C), and that enrichment of the Hmga2 promoter by Hmga2 ChiP-seq was significantly reduced in KCs cultured with 200 nm panobinostat compared with DMSO (Figure 2D). Thus, the HDACi, Panobinostat, significantly decreased transcription of Hmga2, by preventing binding of Hmga2 to it's own promoter.

We next evaluated the effect of panobinostat on the intracellular protein distribution of Hmga2 in cultured KCs. Consistent with the transcript levels, Hmga2 protein levels were significantly downregulated one day after treatment. Interestingly, aside from the effect of Panobinostat on Hmga2 transcription, Panobinostat also caused Hmga2 protein to be re-located from the nucleus to cell body of cultured KCs (Figure 2E, 2F). The results were similar for unsorted KCs (Supplementary Figure 3C–3E). To further investigate the role of panobinostat in the cellular distribution of Hmga2, we performed skin organ cultures. In uncultured skin, distribution of Hmga2 was in the cellular membrane (Figure 2G). However, two days after skin organ culture, Hmga2 redistributed into the entire cell (Figure 2H–2Hiii), similar to what we demonstrated for isolated KCs. However, when Panobinostat in stead of DMSO was added to the skin organ cultures, Hmga2 remained excluded from the nucleus of epithelial cells (Figure 2I–2Iiii), in line with what we demonstrated for isolated KCs in culture.

We also treated mice with papillomas created using a DMBA/TPA protocol with panobinostat [28]. Panobinostat was given i.p. at 15 mg/kg 3 times a week (M/W/F) for 3 weeks when 1 mm benign papilloma formed. Hmga2 translocation from the cytoplasm to the nucleus in the epidermis was also inhibited by panobinostat. More than 50% of the cells in the papillomas of vehicle control treated mice contained nuclear Hmga2 (Figure 2J, 2K), while in panobinostat treated mice, there were either no or lower levels of Hmga2 protein in the nucleus, and in some cells Hmga2 was detected in the membrane (Figure 2L and 2M–2Mi). In addition, panobinostat affected papilloma growth: although the number of papules and small size papillomas (1–2 mm) was not different between mice treated with panobinostat and vehicle control, no medium size (3–5 mm) papillomas were formed in mice treated with panobinostat (Figure 2N, Supplementary Figure 5A–5C). In addition, KI67 positive cells in papillomas were significantly decreased in the panobinostat treated group (Supplementary Figure 6A and 6B), demonstrating that Panobinostat also prevented translocation of Hmga2 *in vivo* in developing tumors, which was associated with a decreased proliferation of transformed cells.

### Panobinostat-mediated inhibition of Hmga2 translocation occurs via ROCK1

**Figure 3:**
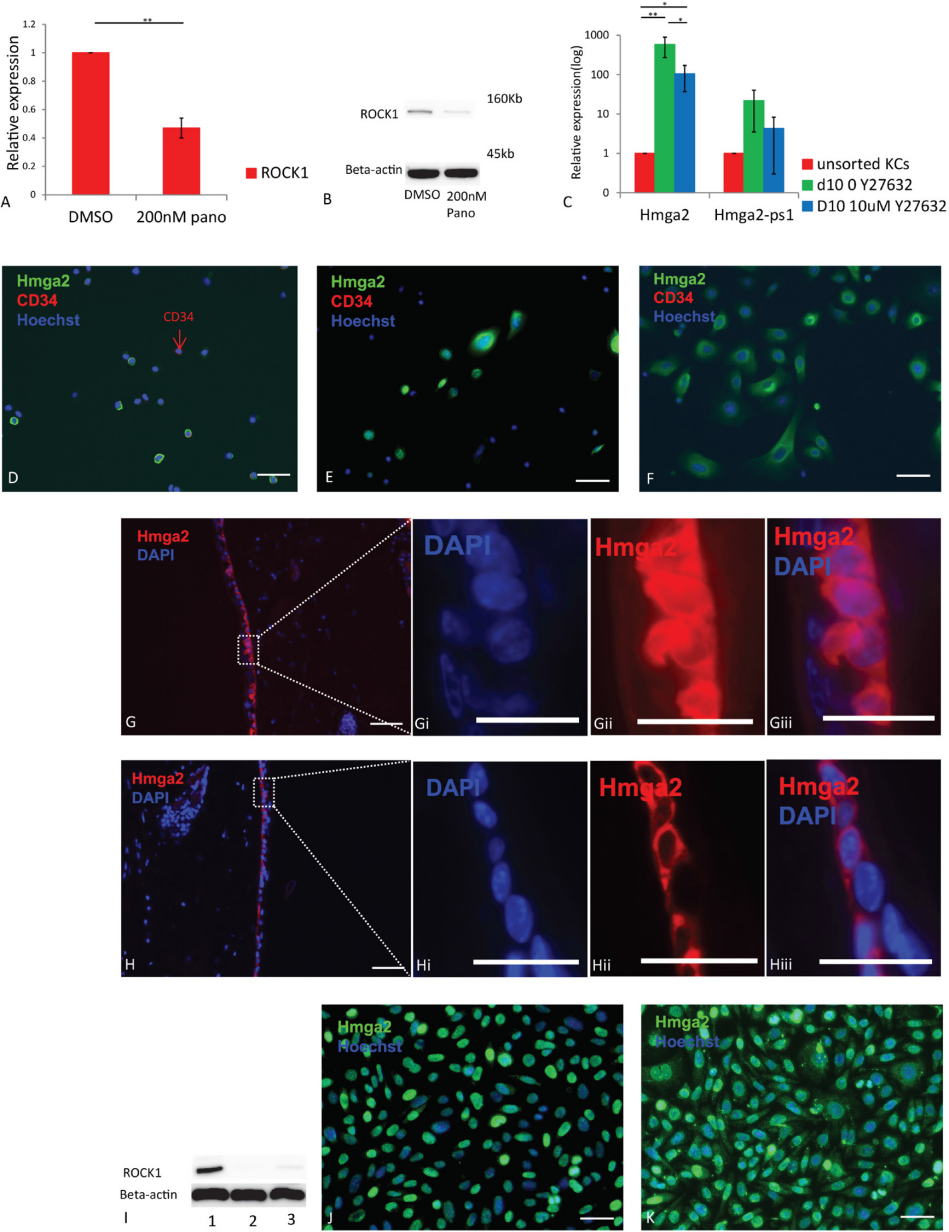
The inhibition of Hmga2 translocation is through ROCKs (**A**) ROCK1 mRNA levels were assessed by qRT-PCR in KCs treated with 200 nM panobinostat. (**B**) Western Blot analysis of ROCK1 expression in KCs after treated with panobinostat. (**C**) Hmga2, Hmga2-ps1 mRNA levels were assessed by qRT-PCR in unsorted KCs, day 10 cultured KCs without Y27632 and day 10 cultured KCs treated with 10 uM Y27632. (**D**–**F**) Unsorted, day 10 cultured KCs without Y27632 and day 10 cultured KCs treated with Y27632 were fixed, and subjected to immunofluorescence using Hmga2 and CD34 antibodies. Nuclei were stained using Hoechst33342, Hmga2 green, CD34 red (*n* = 3). (**G**) KCs were transduced with either *ROCK1* (sh-ROCK1-3 and sh-ROCK1-5) or mock shRNA lentiviral vectors. ROCK1 protein expression was examined by immunoblot. (**H** and **I**) Hmga2 expression in the KCs with mock and ROCK1 knock-down. Nuclei were stained using Hoechst33342, Hmga2 green (*n* = 3). (**J**, **K**) *Ex vivo* cultured skin tissue treated with medium (J) and 100 μM Y27632 (K) were fixed, and subjected to immunofluorescence using Hmga2 antibodies. Nuclei were stained using DAPI, Hmga2 red. Ji–Jiii. Higher magnification showing the expression of Hmga2 in the nucleus and cytoplasm. Ki-Kiii. Higher magnification showing the expression of Hmga2 in the cytoplasm. The scale bar size 50 mm. *P < 0.05. **P < 0.01.

To understand how panobinostat inhibited Hmga2 translocation, we assessed the effect of panobinostat treatment on ROCK1 mRNA and protein levels as inhibition of ROCK can enhance let-7 repression (14). Treatment of KCs with 200 nM panobinostat significantly reduced both transcripts and protein levels of ROCK1, suggesting that Panobinostat may at least in part affect Hmga2 via inhibiting ROCK1 expression (Figure 3A–3B). As Y27632 is a ROCK1 inhibitor, we treated cultured KCs with or without Y27632. RT-qPCR analysis showed significantly lower Hmga2 transcript levels in KCs cultured for 10 days with Y27632 compared to control no-Y27632 group (Figure 3C). Moreover, Hmga2 protein, present in the nucleus and cell of unsorted KCs, cultured for 10 days without Y27632, remained outside of the nucleus in the majority of KCs treated with Y27632 (Figure 3D–3F). Similar results were seen for passage 12 KCs (Supplementary Figure 7A–7C). Consistently, when 100 uM Y27632 was added to skin organ culture, Hmga2 did not relocate from the cell membrane in fresh organs to the whole cell after two days of culture in the presence of Y27632 (Supplementary Figure 8 without treatment; Figure 3G–Giii with medium; Figure 3H–Hiii with medium+Y27632), providing functional evidence for the role of ROCK-1 in Hmga2 intracellular localization. To further prove the role of ROCK1 in Hmga2 translocation, we created ROCK1-knock-down (KD) KCs (Figure 3I). Immunostaining demonstrated that Hmga2 was found in the entire cell in most KD cultured KCs while only in the nucleus of control cultured KCs (Figure 3J, 3K), providing genetic evidence that ROCK1 is involved in relocalization of Hmga2 from the cell cytoplasm/membrane to the nucleus.

## DISCUSSION

Increasing evidence implicates both molecular changes and protein mislocalization as contributing to carcinogenesis. Hmga2, which is widely expressed in undifferentiated cells during embryogenesis, is expressed only in the mesenchyme in adults [9]. In postnatal life, aside from playing a role in stem cell selfrenewal [16, 17], Hmga2 was shown to play an important role in a variety of malignant tumors [21–26]. Whether Hmga2 is involved in skin cancer development has not yet been addressed.

We here demonstrate that Hmga2 is expressed in non-transformed keratinocytes throughout the skin (and esophagus), located to the cell membrane. We demonstrated further that upon induction of cell proliferation *in vitro*, and during the development of cSCC, Hmga2 protein relocates to the nucleus, where it activates expression of Hmga2 protein by binding to and activating the Hmga2 promoter. This process can be inhibited by the HDACi, panobinostat, as well as by inhibiting ROCK1.

High levels of Hmga2 have been observed in several mesenchymal tumors and various human carcinomas [29], where it is expressed mainly detected in the cell nucleus. In addition, during development, Hmga2 is also chiefly located in the nuclear compartment. Here we demonstrate that not only in postnatal quiescent KCs, Hmga2 can be found, not in the nucleus but in the cell membrane, but also we observed increased expression of Hmga2 as well as translocation of Hmga2 to the nucleus upon induction of KC cell proliferation. This was observed using isolated KCs *in vitro*, where Hmga2 was fully nuclear after long-term culture and in skin organ cultures, where Hmga2 was found throughout the cells 2 days following culture. Importantly, we also demonstrated that and upon transformation to skin papillomas and carcinomas *in vivo*, Hmga2 initially relocated throughout the cell with still some cell membrane associated Hmga2 in papillomas, but relocated to the nucleus in carcinomas. The role of Hmga2 in the cell membrane is currently not known. However, our studies indicate that relocation of Hmga2 to the nucleus, which is associated with increased cell proliferation (Ki67 positive cells) (Supplementary Figure 6A and 6B).

High-level expression of Hmga2 in mesenchymal and epithelial cancer cells [31] has been linked to rearrangements and mutations of HMGA caused by chromosomal translocations involving the HMG locus [31]. To determine the mechanism underlying the increased Hmga2 expression levels in proliferating and transforming KCs, we assessed that Hmga2 has an autoregulatory function by promoter-luciferase assay and ChIP-PCR. Our data suggest that when KCs start to proliferate, Hmga2 translocates to the cell nucleus where it causes increased Hmga2 expression by directly binding and activating it's own promoter.

In line with the description that Hmga2 may play a role in hair follicle stem cell selfrenewal [16], we found higher levels of Hmga2 transcripts in CD34+/CD49f+ cells isolated from the keratinocyte fraction of the skin. However, we could not detect Hmga2 protein in the CD34+ fraction. This discrepancy between transcript and protein levels in hair follicular stem cells might be explained by the fact that the let-7 was significantly higher expressed in CD34+/CD49f+ cells compared with CD34+/CD49f- depleted fraction. This is consistent with the fact that there are seven let-7 binding sites in the Hmga2 3′-UTR, and that during development expression of let-7 is inversely correlated with expression of Hmga2, likely due to post-transcriptional modification of Hmga2 protein via let-7 miRNA [12].

Aside from the transcriptional regulation of Hmga2 by Hmga2 itself, as we demonstrate here, and the post-transcriptional regulation by let-7. We also demonstrate that the HDACi, panobinostat, can also decrease the autoregulatory activation of Hmga2 expression. Although inhibition of Hmga2 expression by panobinostat was shown to be mediated by affecting levels of let-7 in the liver cancer cells [15], we did not find changes in let-7 expression in the KCs described here (Supplementary Figure 3B). White, et al., found that Hmga2 was dispensable for cSCC formation as tumors developed in Hmga2 KO mice also overexpressing KRas^G12D^. In their study they found that loss of Hmga2 was associated with increased levels of Hmga1, and suggested that Hmga1 could compensate Hmga2 loss [32]. Differences between the two studies might be the dosage of DMBA/TPA used to create the mouse model. Hmga1 expression was unchanged in our study following treatment with panobinostat, suggesting that Hmga1 may not compensate from the depressed levels of Hmga2 induced by panobinostat (Supplementary Figure 1D).

A final question we addressed is the mechanism by which treatment with panobinostat prevented translocation of Hmga2 to the nucleus, and hence the auto-regulation of Hmga2. Yoshikawa et al., described that inhibition of ROCK can enhance let-7 repression [14], while Zhao et al., demonstrated that and ROCK1 can induce ERK nuclear translocation [33]. We here demonstrated that panobinostat inhibits ROCK1 expression, and that inhibition of ROCK by the Y27632 inhibitor could prevent Hmga2 relocation to the nucleus in KCs and skin organ culture in a concentration dependent manner. Further using ROCK1-KD cultured KCs, Hmga2 expression was found the entire cell, but not exclusively in the nucleus. This suggested that the inhibition of panobinostat on Hmga2 is at least in part through ROCK1.

Overall, these findings demonstrated that Hmga2 is expressed in the membrane of KCs, but translocates to the nucleus upon culture of isolated KCs or KCs in the context of skin organ culture. In addition, Hmga2 translocates to the nucleus during malignant transformation. Hmga2 can in these setting activate it's own receptor, causing elevated levels of Hmga2. All these processes can be inhibited by treatment of KCs, skin organ cultures or of DMBA/TPA treated mice with the HDACi, panobinostat, which prevents binding of Hmga2 to it's promoter and prevents relocation of Hmga2 to the nucleus, the latter in part by inhibiting Rock. These studies demonstrate therefore a prominent role of Hmga2 in cSCC development, even if further studies will be needed to understand the function of Hmga2 in the membrane of quiescent KCs and the complete mechanism of Hmga2 translocation. These studies also suggest a possible role for HDACi in treatment of cSCC.

## MATERIALS AND METHODS

### Keratinocyte cell culture, transfection and sorting

Primary KCs were harvested from the backs of adult C3H mice at 54 ± 2 days and KC cell lines arising from these cultures were maintained in Morris II medium [34]. Cell lines were periodically tested for *Mycoplasma*. No other authentication was performed. For transient transfection in HEK293 (ATCC, cat no.SD-3515,) and in the KC cell line, cells were transfected using VIROMER RED (Lipocalyx GmbH, Germany). For ROCK1 knockdown in KCs, cells were transfected with pooled shRNA reagent (All shRNAs were obtained from the BioMedical Genomics Center at The University of Minnesota). The negative control vector was the pLKO.1 vector backbones that has no hairpin insert. Primary KCs were stained with antibodies to CD34 (rat anti-mouse CD34-FITC, BD Pharmingen); rat anti-mouse CD49f-PE [(alpha-6 integrin), BD Pharmingen] and stem cell fraction (CD49f+CD34+) and stem cell depletion fraction (CD49f +CD34−) were sorted using Fluorescence Activated Cell Sorting (FACS) as previously described [28].

### RNA extraction and quantitative RT-PCR

RNA was isolated using the RNeasy Micro-kit (Qiagen), and DNase treatment with the Turbo DNAse kit (Ambion). cDNA synthesis was performed from 1 μg of RNA with Superscript III First-Strand synthesis system (Invitrogen). Quantitative Real time PCR (qRT-PCR) was performed using the Platinum SYBR green qPCR Supermix-UDG (Invitrogen) and the Eppendorf realplex/ABI 7000 (Eppendorf, Applied Biosciences). Relative gene expression was calculated by the 2(− DDCt) method compared to control group, using GAPDH as housekeeping gene. The list of primers used can be found in Supplementary Table 1.

### Immunofluorescence assay

KCs were fixed using 10% Neutral Buffered Formalin (Fisher Scientific) for 15 min at room temperature (RT). Permeabilization was done for 10 min using a phosphate buffered saline (PBS) containing 0.1% Triton X-100 (PBST) (Acros Organics). PBST, containing 5% normal horse serum (Jackson), was used for blocking for 30 min at RT. The cells were then incubated with the mixture of primary antibodies or appropriate isotype control antibodies diluted in PBS containing 5% horse serum, and incubated overnight at 4°C After 3 washes in PBS, the cells were incubated with the mixture of respective Alexa dye conjugated secondary antibodies (Invitrogen) for 30 mins and Hoechst333425 dye (Sigma) for 5 min at RT. The list of primary and secondary antibodies used can be found in Supplementary Table 2. To enumerate the percentage of cells that stained positive, cells were imaged using a Zeiss Axioskop microscope and AxioVision Version Rel 4.0 software was used to quantify the number of positive cells in 5 to 10 random areas per slide and per condition.

Skin tissues were collected and fixed with 10% formalin for at least 24 hours and then put into 70% ethanol at 4°C. Paraffin-embedded skin sections were rehydrated in a series of ethanol solutions and antigen-retrieved with antigen unmasking solution (H3300, Vector Laboratories, Inc. Burlingame, CA 94010) in a microwave oven for 5 minutes. The slides were then incubated with the rat primary antibody, overnight at 4°C, followed by washing and subsequent incubation in secondary antibody (Invitrogen, Alexa) in a 1:500 dilution. The slides were washed and incubated in the VECTASHIELD (H-1200, Vector Laboratories).

### Plasma membrane extracts

Plasma membrane extracts of unsorted KCs were prepared using a plasma membrane protein extraction kit (Biovision). Cells were harvested at 5–10 × 10^8^ in cold PBS, spun down, washed once in cold PBS and frozen. Homogenization (with Dounce homogenizer) and plasma membrane protein extraction were performed according to the manufacturer’s instructions using buffers included in the kit. Plasma membrane fraction was tested for the presence of plasma membrane markers such as Na-ATPase.

### Western blotting

Cells were harvested and frozen at −80°C. Protein extracts were prepared in a RIPA buffer (Sigma) plus proteinase inhibitor and phosphatase inhibitor (both from Roche Diagnostics). Equal amounts of protein lysate (20 to 40 mg) were separated on NuPAGE gels (Invitrogen). Proteins were transferred onto Immobilon-P transfer membranes (Millipore Corp.) and analyzed by Western blotting using antibodies recognizing the following proteins: Hmga2, Na-K ATPase, Rock1, Histone H3, Acetyl-Histone H3 and beta-actin were purchased from Cell Signaling Technology, another Hmga2 Ab from R&D. Images were visualized with an enhanced chemiluminescence detection kit (ECL-Plus; Amersham Pharmacia Biotech) and the ImageQant LAS 4000 imaging system (GE Healthcare life Sciences). Results of Western blot analyses were representative of two to four independent experiments.

### Luciferase reporter assay

Luciferase reporter plasmids driven by the Hmga2 promoter (named pHmga2-Luc) were constructed by inserting the −800 to +197 Hmga2 promoter sequence [11] into the pGL3 luciferase reporter vector or a combination of pLL3.7-Hmga2 expression constructs. The promoter and Hmga2 CDS was confirmed by sequencing. Mouse KCs were used to assess the promoter activation. After mouse KCs (250,000/cm2) were treated with DMSO and 200 nM panobinostat, and then transfected with each of the reporter vectors together with pRL-tk plasmid (Promega) (at 1/10 of the DNA amount for the test vector) and Renilla luciferase as an internal control for possible variation in transfection efficiency. Cells were harvested 48 h after transfection with VIROMER red transfection reagent (Lipocalyx), and firefly and Renilla luciferase activities in the lysates tested using the Dual-Luciferase Reporter Assay System (Promega) on a Luminoskan Ascent (Labsystems). The ratio between Firefly and Renilla luciferase activity was obtained for each sample. Relative luciferase units were calculated via normalization of each of the ratios for all groups by the average ratio for the promoterless group.

### Flow cytometric analysis

Surface and intracellular staining with unsorted KCs and passage 12 KCs were performed. Flow cytometric analyses were performed with Flowjo (Tree Star). Single cells from unsorted KCs, passage 12 KCs and permeabilized passage 12 KCs by 90% acetone was detected. Cells were stained with the Hmga2 (Cell Signaling Technology) for 90 mins under 4 degree and add Alex488 (0.2 mg/ml, Invitrogen) for another 60min under 4 degree.

### Chromatin immunoprecipitation-qPCR

Chromatin isolation and IP were performed according to the instructions from the Transcription Factor ChIP (Chromatin immunoprecipitation) kit (Diagenode). Chromatin was isolated from nearly 5 × 10^7^ KCs treated with or w/o panobinostat. Antibodies against Hmga2 (Gene Tex) or an isogenic antibody (Santa Cruz) were used at 2 mg per IP reaction. Purified DNA was used as template for qRT-PCR using Platinum SYBR green to amplify the Hmga2 promoter or GAPDH promoter(Supplementary Table S1). The PCR product size was 94 bp (Hmga2 promoter) and 104 bp (Gapdh promoter). The following PCR conditions were used, 3 min at 95°C and 40 cycles of 15 s at 95°C, 45 s at 60°C and 1 min at 95°C.

### Organotypic Skin Culture

Female C3H mice were bred at University of Minnesota, Hormel institute. Cages were changed twice weekly; food and water were available ad libitum. Untreated mice 7 to 9 wk of age were euthanized by CO2 by inhalation followed by cervical dislocation, clipped, depilated with Nair (Carter Products, Division of Carter-Wallace, Inc., New York, NY), and washed with povidone-Iodine (American Sterilizer Co., Erie, PA) and 70% ethanol. The dorsal skin was excised and placed in sterile calcium-and magnesium-free Dulbecco's phosphate buffered saline (Gibco) with 100 mg/liter of gentamicin sulfate (Whittaker M.A. Bioproducts). Each piece of skin was placed in a sterile petri dish (Corning) and the subcutaneous fat and muscle were removed by scraping with a scalpel with a number 22 blade. Pieces of 1 mm^2^ were cut with a new sharp blade (a clean cut with very sharp blade seemed to be important for subsequent epidermal outgrowth) and placed onto 100-mm Corning tissue culture dishes (Corning biocat) and incubated with Morris II medium plus DMSO or panobinostat.

### Mice

Female SKH-1 mice (6 weeks old) were purchased from Charles River Laboratories (Wilmington, MA). The animals were housed five per cage at 22 ± 10°C and 50 ± 10% relative humidity and subjected to a 12 h light/ 12 h dark cycle in the College of Pharmacy animal facility. They were acclimatized for 1 week before use and provided food and water ad libitum. Non-melanoma skin cancer was induced by one time treatment of Dimethylbenz(a)anthracene (DMBA; 100 nmol in 200 microliters of acetone) to the dorsal skin. Upon treatment, the mice were placed in disposable cages where they remained for 1 week. During this time, the mice continued to live in SPF housing. One week later, mice received twice weekly treatments (Tuesday/Thursday) with the tumor promoter 12-O-tetradecanoylphorpol-13-acetate (TPA: 5 nmol in 200 microliters of acetone) for up to twenty weeks. This was followed by treatment with panobinostat which was purchased from Selleck Chemicals (Houston, TX, USA) at a concentration of 1.5 mg/mL or vehicle (2% DMSO, 2% Tween 80, 48% PEG 300, 48% water) when papilloma size reaches to 1–2 mm and administered 3 times a week [Monday/Wednesday/Friday (M/W/F)] in a volume of 10 mL/kg by intraperitoneal (i.p.) injection.

### Statistics

Results are expressed as mean ± SEM. Statistical significance was determined by student's *t*-test.

## SUPPLEMENTARY MATERIALS FIGURES AND TABLES


