# Supplementary material for: Hmga2 translocation induced in skin tumorigenesis
Source: Oncotarget. 2017 Mar 16;8(18):30019–29. doi: 10.18632/oncotarget.16272 (PMC5444722; doi:10.18632/oncotarget.16272)
Supplement: Supplementary file 1 [file oncotarget-08-30019-s001.pdf]

Hmga2 translocation induced in skin tumorigenesis

Supplementary Materials

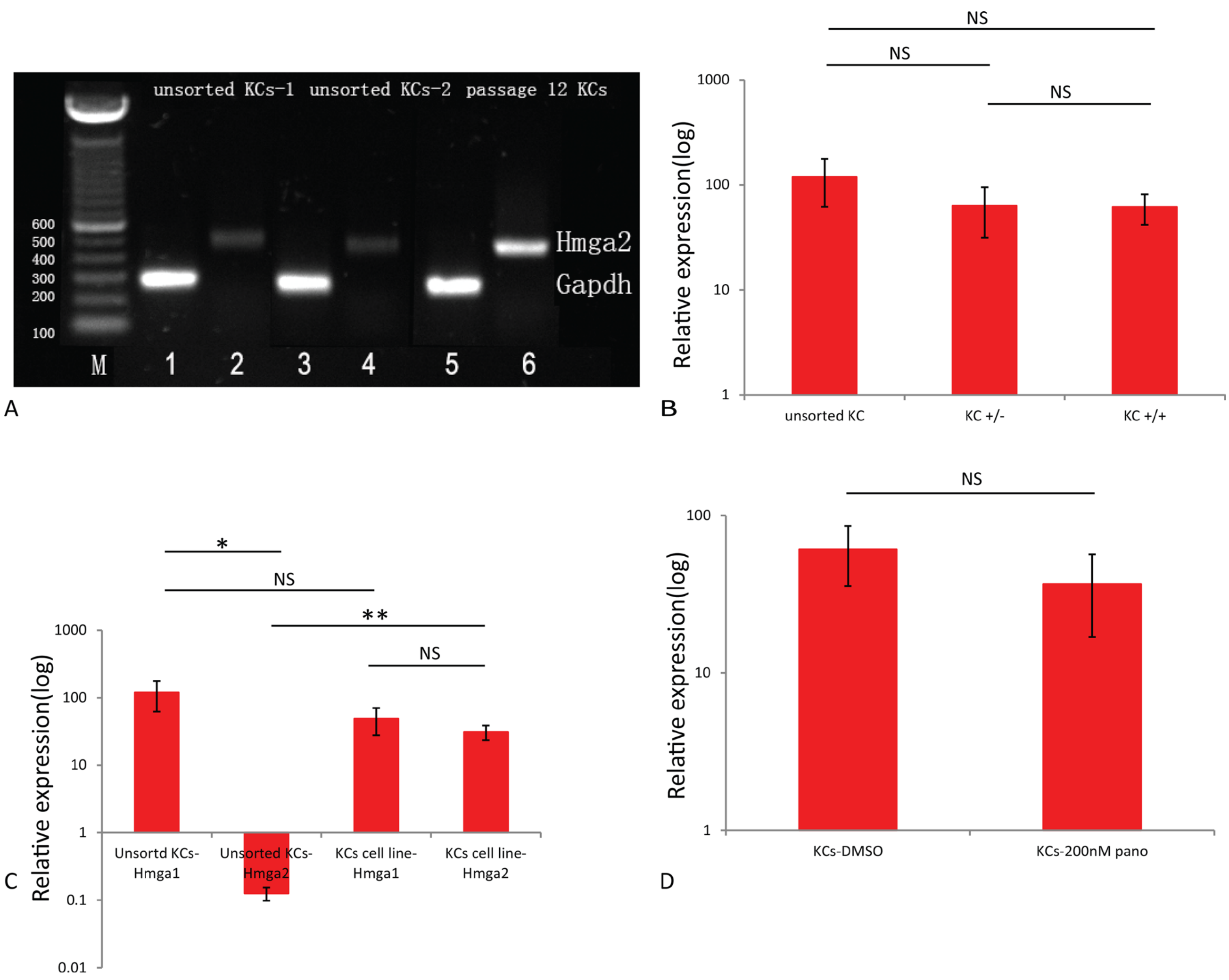

Supplementary Figure 1: showed Hmga2 and Hmga1expression in KCs. Hmga2 and Hmga1 mRNA expression in KCs. (**A**) Agarose gel electrophoresis of RT-PCR products amplified using Gapdh primer (lanes 1, 3, 5) and Hmga2 primer (lanes 2, 4, 6) for unsorted KCs and passage 12 KCs. The Hmga2 primer sequence from cancer res [35] (Supplementary Table 1). Hmga2 exon size (480 bp), Gapdh exon size (281bp). (**B**) Hmga1 mRNA expression in unsorted KCs, CD34−CD49f+ and CD34+CD49f+. (**C**) Hmga1 and Hmga2 mRNA expression were assessed by qRT-PCR in unsorted KCs and cultured KCs. D. Hmga1 mRNA expression were assessed by qRT-PCR in KCs treated with DMSO and 200nM panobinostat (*n* = 3 or 5).

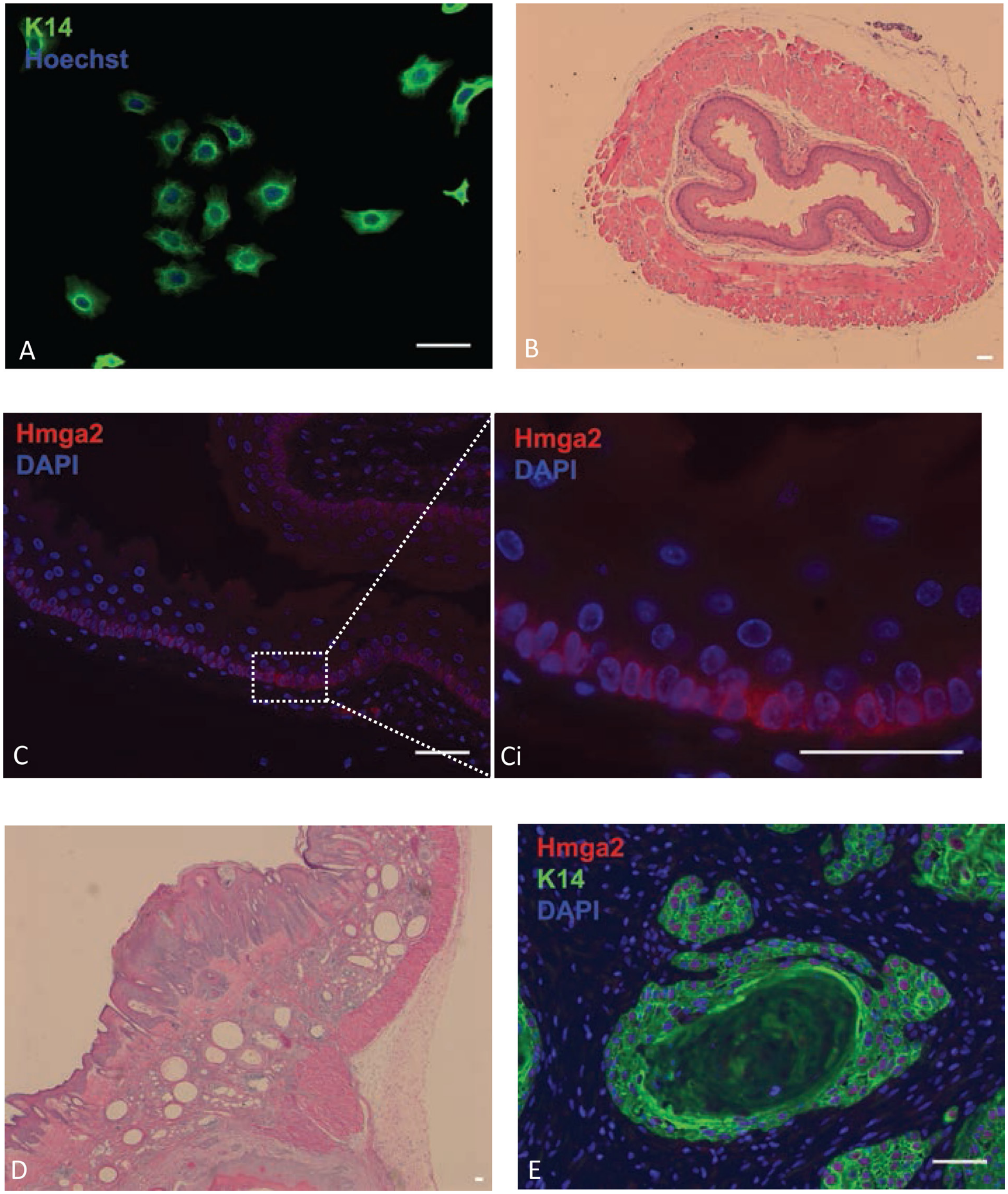

Supplementary Figure 2: showed K14, Hmga2 expression in KCs, esophagus and skin carcinoma tissue. Expression of K14, Hmga2 in KCs, esophagus and skin carcinoma tissue. (**A**) Representative image of K14 expression in KCs at passage 12 was shown. (**B**) H&E staining for mouse esophagus tissue. (**C**) Representative image of Hmga2 expression in esophagus was shown. Ci. Higher magnification showing the expression of Hmga2 in membrane. (**D**) H&E staining for skin carcinoma. (**E**) Representative image of Hmga2 and K14 expression was shown. DNA (blue), Hmga2 (red) and K14 (green). Scale bar, 50 μm.

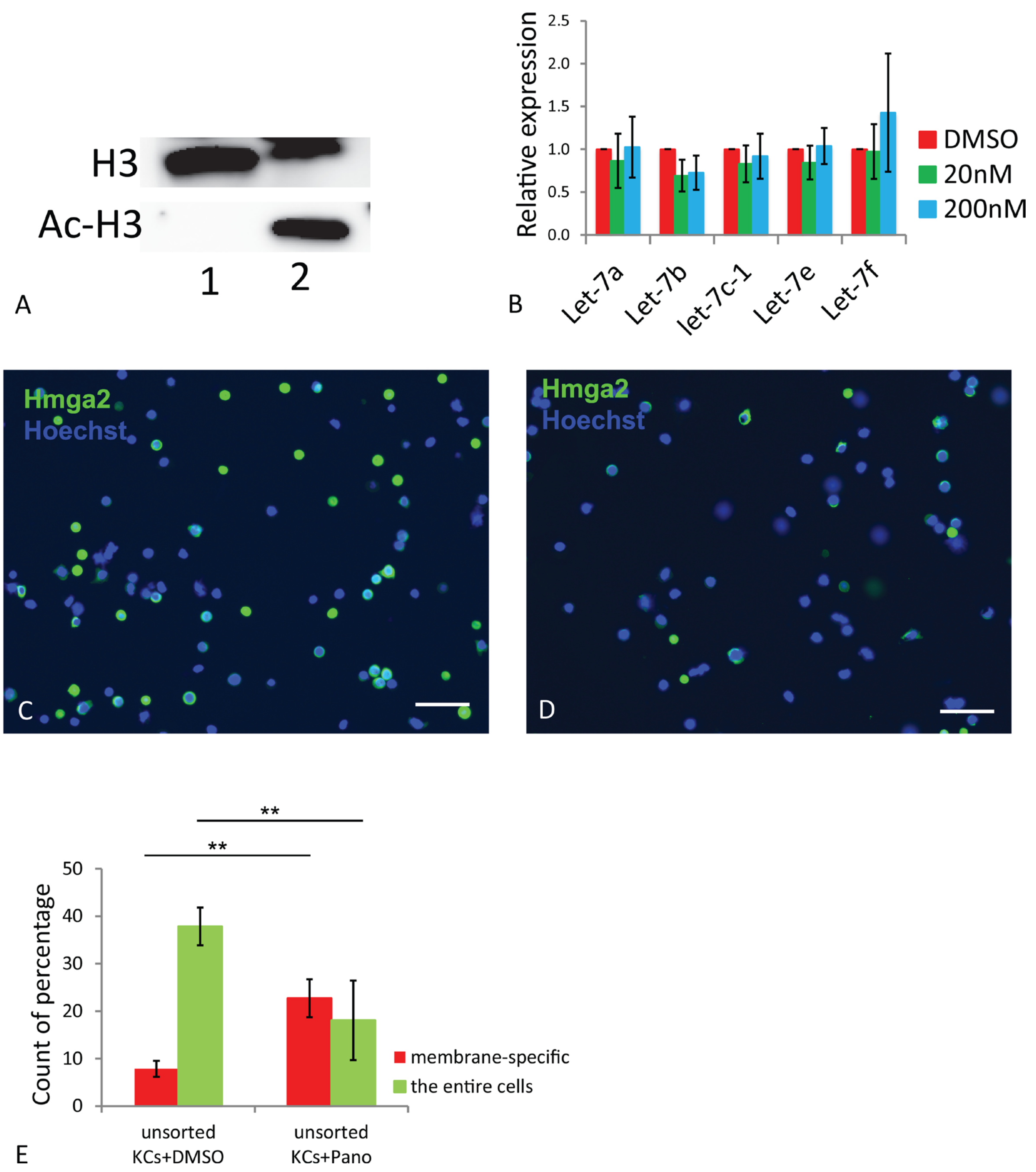

Supplementary Figure 3: showed Histone 3, acetyl-Histone 3, let-7 and Hmga2 expression in KCs after panobinostat treatment. Histone 3, acetyl-Histone 3, let-7 and Hmga2 expression in KCs after panobinostat treatment. (**A**) The protein expression of Histone H3 and acetyl histone H3 for KCs treated with DMSO (lane 1) or 200 nm panobinostat (lane 2) for one day. (**B**) let-7 family was assessed by qRT-PCR in KCs treated with DMSO, 20nM and 200nM panobinostat. (**C–D**) Representative image of Hmga2 expression in unsorted KCs treated with DMSO (C) and 200nM panobinostat (D) for one day. (**E**) Percentage of cell count for hmga2 expression in the membrane and entire cells in the unsorted KCs treatment with DMSO or 200nm panobinostat one day. Shown in the images are DNA (blue) and Hmga2 (green). Scale bar, 50 μm $^{**}P < 0.01$.

A

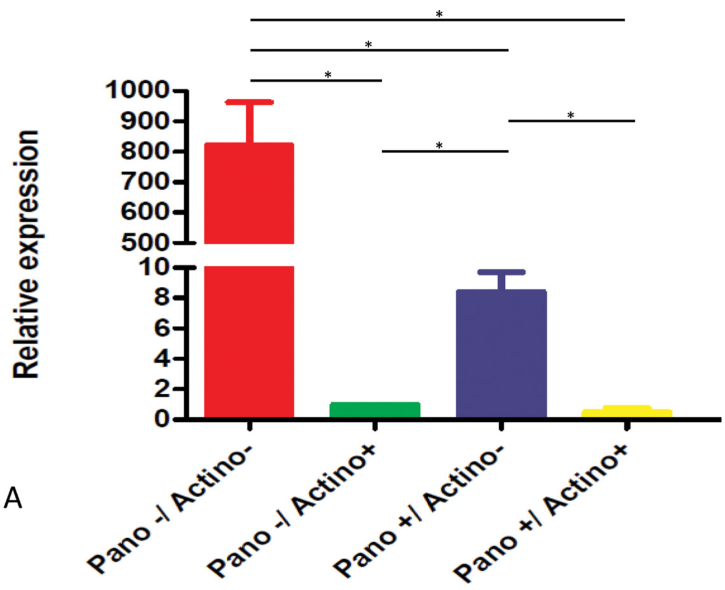

Supplementary Figure 4: showed the inhibition of Hmga2 transcription by panobinostat and actinomycin D. (**A**) Panobinostat repressed transcriptional activity of the endogenous Hmga2 gene other than the inhibition of actinomycin D. Hmga2 mRNA was assessed by qRT-PCR in KCs treated with actinomycin D and 200 nM panobinostat. *$P < 0.05$.

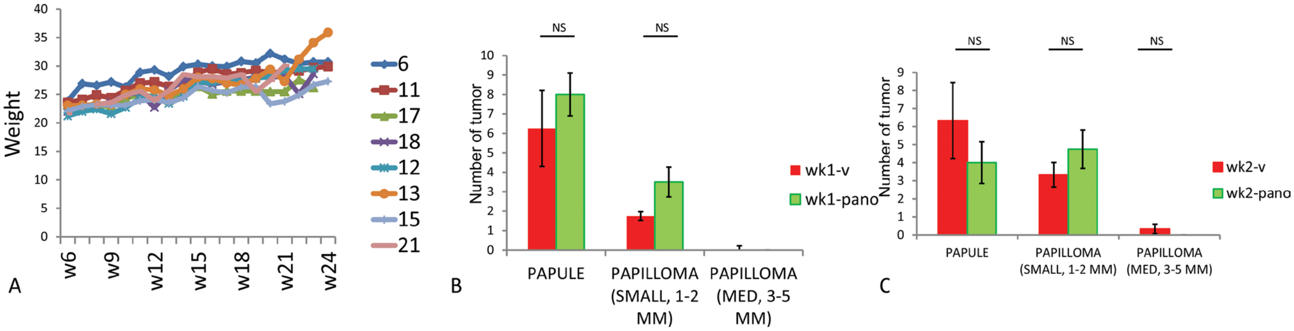

Supplementary Figure 5: showed the weight and the number of papilloma in SKH mice treated with vehicle and panobinostat. The body weight and the number of papilloma in SKH mice treated with vehicle and panobinostat. (**A**) The body weight was measured for SKH mice treated with vehicle and panobinostat. (**B**–**C**) The number of papule, small papilloma (1–2 mm) and medium papilloma (3–5 mm) were counted in SKH mice after treated vehicle and panobinostat at week1 and week2. NS, no significant. *$P < 0.05$.

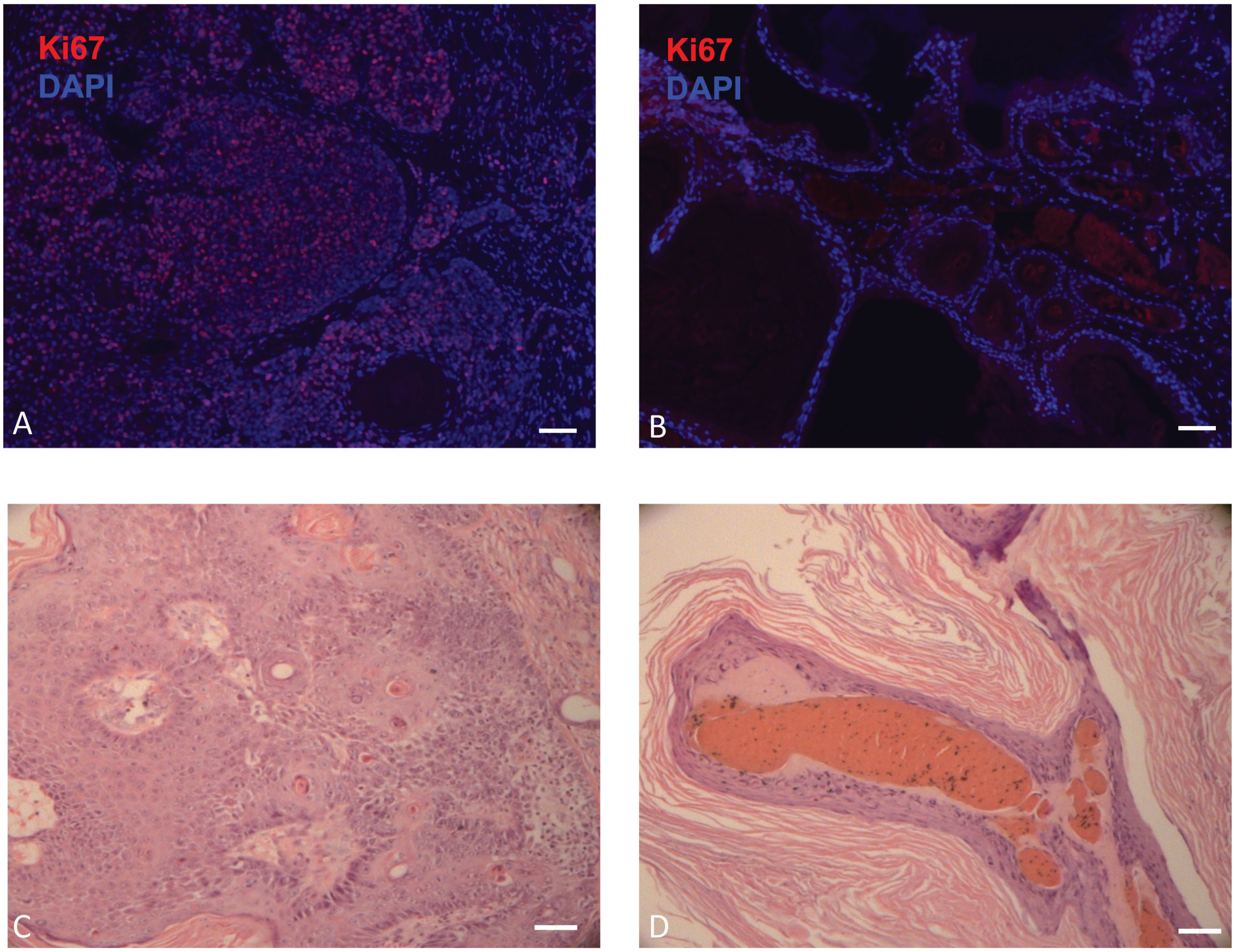

Supplementary Figure 6: showed Ki67 expression in the skin tissue from mice treated with vehicle and panobinostat. Ki67 expression in the skin tissue from mice treated with vehicle and panobinostat. Representative image of Ki67 expression in mice treated with vehicle and panobinostat. (**A**) Papilloma tissue from the mouse treated with vehicle; (**B**) papilloma tissue from the mouse treated with panobinostat. Shown in the images are DNA (blue) and Ki67 (red). (**C**–**D**) H&E staining for papilloma. (C) vehicle treatment, (D) panobinostat treatment. Scale bar, 50 μm.

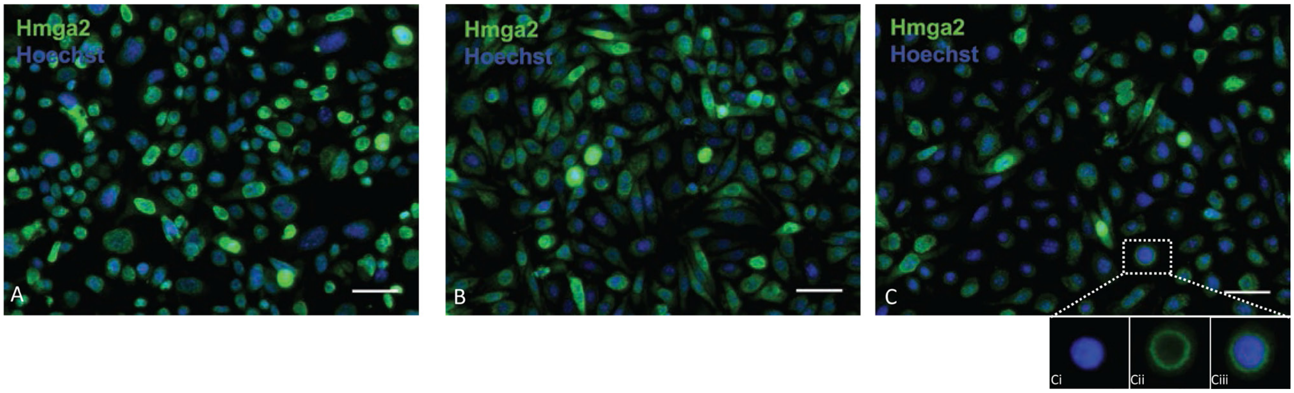

Supplementary Figure 7: showed Hmga2 expression in KCs treated with Y27632. Expression of Hmga2 in KCs treated with Y27632. (**A**–**C**) Representative image of Hmga2 expression in KCs treated with medium (A), 10 μM Y27632 (B) and 100 μM Y27632 (C) for four days. Ci-Ciii. Higher magnification showing the expression of Hmga2 in the membrane after treated with Y27632. Shown in the images are DNA (blue) and Hmga2 (green). Scale bar, 50 μm.

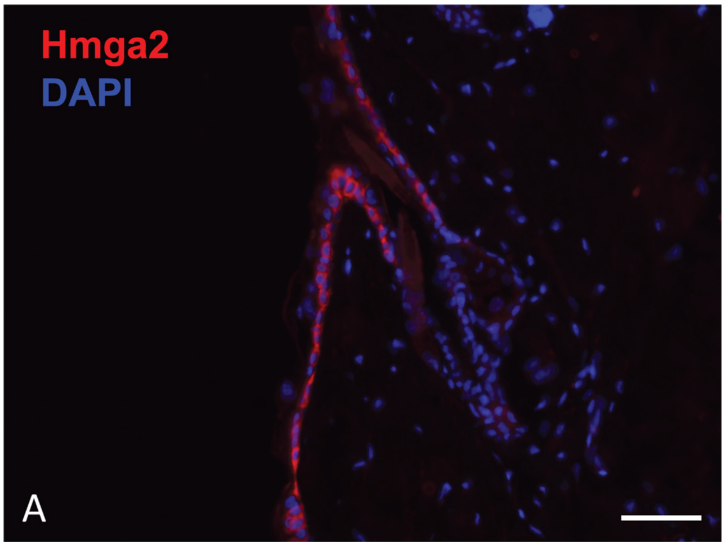

Supplementary Figure 8: showed Hmga2 expression from skin organ culture without treatment. Hmga2 expression in skin organ culture without treatment. A. Representative image of Hmga2 expression from mouse skin organ. DNA (blue) and Hmga2 (red). Scale bar, 50 μm.

Supplementary Table 1: List of primers used in this study for RT-PCR and qRT-PCR

NAME	Sequence
GAPDH for	CATCCATGACAACTTTGGCA
GAPDH rev	CATCATACTTGGCAGGTTTCTC
HMGA2 for	CAGCCCAGAAGAAAGCAGAG
HMGA2 rev	GCAGGAAGTAGAAAGACCGT
HMGA1 FOR	GCCCCACACATAGAGAAGGA
HMGA1 REV	CAGAGGACTCCTGGGAGATG
HMGA2 for from Cancer research, 1999	ATATAAGCTTGGTACCGGTAGAGGCAGTGG
HMGA2 rev from Cancer research, 1999	ATATAAGCTTACCCCGCAGGAAGTAGAAAG
HMGA2 Pseudo gene for	AAAGGATATGGGGGAAGTGG
HMGA2 Pseudo gene rev	CTGAGAGGAGAGGCACAACC
CD34 for	CTAGTTGTGAGGAGTTTAAGAAGG
CD34 Rev	CCAAGACCATCAGCAAACAC
ROCK1 for	GATTGTTTGCTGGATGGATTGG
ROCK1 rev	CCTCTGCCGATTACCTTTACC
Hmga2 promoter for	CTACCTCCGCCACCCACT
Hmga2 promoter rev	GGCTGCCAAAAAGAGAGAGG
Gapdh promoter for	AGAGAGGGAGGAGGGGAAAT
Gapdh promoter rev	GCCCTGCTTATCCAGTCCTA
universal reverse primer	GTGCAGGGTCCGAGGT
mm-U6	TGGCCCCTGCGCAAGGATG
mm-miR-let 7a-2	TGAGGTAGTAGGTTGTATAGTT
mmu-let-7b	TGAGGTAGTAGGTTGTGTGGTT
mmu-let-7c-1	TGAGGTAGTAGGTTGTATGGTT
mmu-let-7e	TGAGGTAGGAGGTTGTATAGTT
mmu-let-7f-1	TGAGGTAGTAGATTGTATAGTT
sh-ROCK1-2	TRCN00000022899
sh-ROCK1-3	TRCN00000022900
sh-ROCK1-4	TRCN00000022901
sh-ROCK1-5	TRCN00000022902
sh-ROCK1-6	TRCN00000022903

Supplementary Table 2: List of antibodies used in this study for IF, WB, FC and ChIP

primary Antiboy	company	catlog	original	dilution	Application	2nd antibody	dilution	isotype
Hmga2	CST	8179	rabbit	1:200	IF,Wb, FC	Invitrogen, Alexa Fluor 594, A11012	1:500	normal rabbit IgG, SC-2027
Hmga2	R&D	AF3184	goat	1:20	IF	R&D,anti-Goat IgG, NL001	1:200	normal goat IgG, SC-2028
CD34	BD	553731	rat	1:100	IF	Invitrogen, Alexa Fluor 488, A11006	1:500	Rat IgG2a,K, 553927
K14	Biolegend	PRB-155P	rabbit	1:1000	IF	Invitrogen, Alexa Fluor 488, A11008	1:500	normal rabbit IgG, SC-2027
Ki67	abCAM	AB15580	rabbit	1:200	IF	Invitrogen, Alexa Fluor 594, A11012	1:500	normal rabbit IgG, SC-2027
ROCK1	CST	4035	rabbit	1:1000	Wb	SC-2004	1:3000	
Histone H3	CST	4499	rabbit	1:1000	Wb	SC-2004	1:3000	
Acetyl Histone H3	CST	8173	rabbit	1:1000	Wb	SC-2004	1:3000	
Na-K ATPase	CST	3010	rabbit	1:1000	Wb	SC-2004	1:3000	
Hmga2	CST	5269	rabbit	1:1000	Wb	SC-2004	1:3000	
beta-actin	CST	4970	rabbit	1:1000	Wb	SC-2004	1:3000	
Hmga2	Gene Tex	GTX100519	rabbit		ChIP			normal goat IgG, SC-2028
CD49f	BD	555735	rat		FC			Rat IgG2a,K, 554889
CD34	BD	553733	rat		FC			Rat IgG2a,K, 553929
Hoechst33342	invitrogen	H3570						
